# The predictive mind and the experience of visual art work

**DOI:** 10.3389/fpsyg.2014.01417

**Published:** 2014-12-16

**Authors:** Ladislav Kesner

**Affiliations:** Department of Art History, Masaryk UniversityBrno, Czech Republic

**Keywords:** predictive coding, predictive error minimization, art perception, art experience, affective affordance, reward in art, culturality of vision

## Abstract

Among the main challenges of the predictive brain/mind concept is how to link prediction at the neural level to prediction at the cognitive-psychological level and finding conceptually robust and empirically verifiable ways to harness this theoretical framework toward explaining higher-order mental and cognitive phenomena, including the subjective experience of aesthetic and symbolic forms. Building on the tentative prediction error account of visual art, this article extends the application of the predictive coding framework to the visual arts. It does so by linking this theoretical discussion to a subjective, phenomenological account of how a work of art is experienced. In order to engage more deeply with a work of art, viewers must be able to tune or adapt their prediction mechanism to recognize art as a specific class of objects whose ontological nature defies predictability, and they must be able to sustain a productive flow of predictions from low-level sensory, recognitional to abstract semantic, conceptual, and affective inferences. The affective component of the process of predictive error optimization that occurs when a viewer enters into dialog with a painting is constituted both by activating the affective affordances within the image and by the affective consequences of prediction error minimization itself. The predictive coding framework also has implications for the problem of the culturality of vision. A person’s mindset, which determines what top–down expectations and predictions are generated, is co-constituted by culture-relative skills and knowledge, which form hyperpriors that operate in the perception of art.

## INTRODUCTION

The old notion of perception as unconscious, knowledge-driven inference (Helmholz, 1860/1962) or hypothesis testing (Gregory, 1980), which asserts that the brain actively anticipates upcoming sensory input rather than passively registering it, has now been recast in the terms of contemporary neuroscience, and has recently undergone an unprecedented revitalization. It has been linked to the idea of the Bayesian brain – a probability machine that constantly makes predictions about the world and then updates them based on what it senses. According to predictive coding model of perceptual inference, subjects try to infer the causes of their sensations based on multi-level generative models of the world (Rao and Ballard, 1999; Kersten et al., 2004; Bar, 2009; Friston, 2009; Rauss and Pourtois, 2013). Predictions (priors) about the probable cause of sensory input, generated in higher levels of processing hierarchy, are continuously updated by prediction errors which code mismatches between expected and actual data. Recently, some theorists have further extended the predictive coding framework (or predictive error minimization, PEM) from *brain* to *mind,* applying it to a variety of cognitive mechanisms beyond perception itself (Hohwy, 2013). According to philosopher Clark (2013) the Bayesian approach constitutes the “grand unified theory of mind as perception, action and attention are all in the same business of reducing sensory prediction error resulting with our exchanges with environment” (Clark, 2013, p. 21). As Friston (2013, p. 1330) notes, the higher-order aspects of inference in the brain represent the frontiers of theoretical neurobiology. The main challenge is to link prediction at the neural level with prediction at the cognitive-psychological level and to find conceptually robust and empirically verifiable ways to harness this theoretical framework toward explaining higher-order mental and cognitive phenomena, including the subjective experience of aesthetic and symbolic forms. A step in this direction has been taken in the recently proposed “tentative prediction error account of visual art” (TPEA; Van de Cruys and Wagemans, 2011).

It is the aim of this article to further extend the application of the predictive coding framework to the visual arts. To do this in a productive way requires moving from the rather abstract level of theory formulation to a more detailed discussion of particular instances of response to a work of art, that is, it requires testing the theoretical model against a specific case study. Consequently, the article is organized into three sections. First, I shall articulate some objections to and problems with the current formulation of the prediction error account of art perception. Based on these observations, the second section presents a case study of an encounter with a particular painting in order to expand upon some key some aspects of predictive coding in visual art. I shall focus especially on the problem of the emotional response to a work of art within the PEM framework. Finally, I shall point out further implications of this theoretical model for the question of the social and cultural determination of vision.

**KEYWORD CONCEPTS**

**Bayesian brain**

The notion that perceptual impression results exhaustively from neuronal groups computing probability (P) of a hypothesis (H) given data (D) according to Bayes rule for P(H| D):

P(H|D) = P(H) × P(D|H)/P(D)

The left-hand part of the equation corresponds to *posterior probability*, that is probability that the hypothesis is true given the new data.

P(D|H) refers to the *likelihood of compatibility* of new data with prior hypothesis and

P(H) or *prior* denotes one’s prior expectations about the probability of the hypothesis and drives the interpretation of sensory data.

**Predictive coding**

According to hierarchical predictive coding framework of perceptual inference, brain does not passively register sensory input but actively anticipates it. Neuronal representations at the higher levels of processing hierarchy generate “conditional expectations of perceptual causes” or predictions (priors), based on information in memory and context, about the probable cause of sensory input. These predictions are communicated to lower sensory areas by feedback connections where they are compared with incoming sensory data. Mismatches between expected and incoming data form prediction errors which are passed by feedforward connections to update or change higher-level representations, creating the most reasonable interpretation of incoming sensory input. There are different interpretations of the predictive coding framework and its neurobiological implementation in literature (e.g., Rao and Ballard, 1999; Friston, 2002, 2008; Lee and Mumford, 2003; Knill and Pouget, 2004; Yuille and Kersten, 2006; Summerfield et al., 2006; Bar, 2009; Friston and Kiebel, 2009; Rauss and Pourtois, 2013).

**Prediction error minimization** = minimization of uncertainty about the sensory data.

According to PEM model, brain continuously minimizes its prediction error, that is the discrepancy between predictions (expectations) about the sensory data and the actual data.

I set out from the assumption that it is neither likely, nor indeed desirable, for a prediction coding framework to ultimately form a general biological “theory of art” or aesthetic perception – a holy grail of neuroaesthetics, to which art historians and theorists remain justifiably indifferent. Even if predictive coding could assume the position of a grand unified brain theory (and some doubt it can; see, e.g., Bowers and Davis, 2012; Anderson and Chemero, 2013), there are strong grounds for being skeptical about the prospect of any comprehensive brain theory becoming also a grand biological theory of art and art perception. What such a theory could provide, however, is a framework with which to revisit some long-standing questions in image studies, the psychology of art and art history and out of which further empirical studies of art perception could evolve.

## THE PREDICTION ERROR ACCOUNT OF ART – CURRENT FORMULATION AND ITS CONTRADICTION

The basic tenet of Van de Cruys and Wagemans (2011) model is that a temporary state of unpredictability (or prediction error) is important for the emergence of perceptual pleasure vis-a-vis a work of art. Understanding perception in terms of predictions means that it is possible for perceptual configuration to induce different sequences of affect and to do so partly independently of the particular content of perception (TPEA, 1040). Accordingly, artists are supposed to intentionally create incongruities (perceived as prediction errors) that may not be possible in a natural visual environment, and viewers are able to tolerate and even enjoy the unpredictability because they expect to be surprised in their encounters with art (TPEA, 1041). By delaying prediction confirmation, artists create a positive affect: the viewer quickly runs into incongruities, which presumably generate an arousal aimed at reducing prediction errors. It is this incompatibility (or prediction error) that is the source of some of the emotionality of a work of art. In other words, artists intuitively attempt to strike the optimal balance between predictability and surprise. The mental effort required of a person in order to cope with the prediction error is a condition sine qua non for registering the perceptual pleasure of a Gestalt formation (prediction error reduction). According to Van de Cruys and Wagemans (2011): “Only by using minimal prediction errors painters can ensure that viewers will obtain their reward and not give up prematurely. Final gratification postponed as long as the artist has hidden in the painting enough micro reward the viewer can discover...” (TPEA, 1050).

This concept is appealing for a number of reasons. First, it offers a much stronger explanatory framework than the neuroscientific and psychological models of art experience, which focus exclusively on a bottom–up account of visual processing (Zeki, 1999; Shimamura, 2013) and neglect or downplay the role of the top–down, inferential activity of brain/mind. Second, although not specifically stated by the authors, the model responds to the so-called “dark room problem,” the apparent paradox that, in order to minimize surprise, agents should avoid sensory stimulation altogether and should proceed directly to the least stimulating environment and stay there; they should take up a position in the nearest “dark room” and never move again. Neatly summarized: *avoid surprises and you will last longer*. Predictive coding theorists offer a simple solution to the dark room scenario: prior beliefs render dark rooms surprising. That is, agents that predict rich stimulating environments will find the “dark room” surprising and will leave at the earliest opportunity. The postulate of surprise minimization therefore by no means inhibits subjects from active, exploratory behavior and novelty-seeking, including presumably an aesthetic experience (Friston et al., 2012b; Schwartenbeck et al., 2013).

Third, the model is apparently compatible with some well-established and respected art-historical theories, notably Gombrich’s (1960) theories of the prognostic character of the perception of pictures, the role of the beholder’s share and the viewer’s inferences in perception, and his notion of the artist working through a cycle of scheme and correction. Indeed, Gombrich’s (1960) famous maxim that “[t]o read the artist’s picture is to mobilize our memories and experience of the visible world and to test his image through tentative projections” leaves the door open to the Bayesian brain perspective; an interesting challenge would be to recast Gombrich’s account in the explanatory terms of predictive coding, but this will not be pursued here. Furthermore, it provides scientific footing to some philosophical interpretations of aesthetic experience, most notably Gadamer’s (1975/2004) hermeneutical scenario, which highlights the activity of the perceiving subject vis-à-vis the aesthetic object. Gadamer describes the nature of this exchange as ongoing and dynamic, suggesting that understanding is an open-ended or at least an extended process that does not end the moment the representational content is identified or the information embedded in the work of art obtained, but also includes a more complex response and understanding: “all encounter with the language of art is an encounter with an unfinished event and is itself part of this event...There is no absolute progress and no final exhaustion of what lies in work of art” (Gadamer, 1975/2004, p. 85).

## PREDICTION ERROR OPTIMIZATION VERSUS THE RUSH TO THE OBJECT

Any theory should be measured against empirical findings, in this case on what we know about how people actually interact with works of art. Seen in this light, the main objection to tentative prediction error immediately becomes apparent: the model describes an ideal situation, which represents a distinct minority of actual encounters with art works. This does not invalidate the theory as such, but addressing this discrepancy paves the way to pursuing some crucial aspects of predictive mind in art experience.

When observing people’s reactions in front of works of art in a museum or gallery, one quickly notices that many viewers are content with performing the simple act of recognition, displacing the visual substance of the work as soon as possible with the kind of understanding that evidently does not prompt or entice further viewing. This situation is eloquently captured in an anecdote recounted by Rudolph Arnheim:

*“I remember once watching a teacher with her second-graders approaching a piece of abstract sculpture in a museum gallery. ‘What is this?’ asked the children. The teacher, very unsure herself, went closer and looked at the label. ‘Gift of Mr. and Mrs. Oscar Verlinski,’ she read. The children, satisfied, moved to the next object.*”

(Arnheim, 1992, p. 61)

Besides providing a depressively true account of the nature of many encounters between visitors and works of art in a museum, of the way in which people harvest meaning from works of art, Arnheim’s (1992) observation captures some essential aspect of the process in which viewers make sense of art images. It describes the moment when the visual object, presented to view and soliciting an understanding, gets an “answer.” As a paradigmatic example of an act of translation, or displacement, it highlights the violent and terminal substitution of the visual presence of an art work with a label in the mind’s eye of the viewer. The moment the exhibit becomes the “gift of Oscar Verlinski” in the eyes of the inquiring children, it ceases to be a sculpture, an object endowed with visual and aesthetic interest. In other words, the story neatly describes the psychological reality of surprise minimization in an encounter with an art work. By fixating on identification, the viewer’s interrogation of an object is effectively concluded. And as much as if in the given case the object had been answered “correctly” (as e.g., “abstract sculpture,” or “work by Anthony Caro”). The moment the visible content of the art object is recognized as what it depicts, the viewing is concluded, instead of a series of exchanges between the image and the viewer opening up and ushering in reciprocal play, inviting the viewer into the rich possibilities of dialog ^1^. Very often, moreover, the viewer recognizes the content of the image as its subject – that is, the culturally ingrained capacity for recognizing subjects in pictorial content is grafted onto the biologically ingrained propensity for perceptual identification – and thereby translates or displaces the pictorial meaning ^2^. This mode of grasping a painting or sculpture can be seen as an extension of the evolutionary programmed operation of visual awareness, the role of which is to produce the best current interpretation of the visual scene, in the light of past experience, either our own or of our ancestors, and to make it available to the parts of the brain that plan and execute voluntary motor outputs (Crick and Koch, 1995). The biologically adaptive function of the human visual system, which ontogenetically evolved in the service of assuring physical and social survival, cannot be easily switched off when applied to works of art.

The viewing of art images is thus frequently characterized by a tension between unconscious reaction, a biologically ingrained need on the part of the viewer to understand a visual scene unambiguously, and the fact that stopping at the moment of this initial displacement, “answering” the image in the moment of identification, threatens to annihilate its most valuable asset – the possibility for deeper engagement. One could say that works of art are often victims of the biologically hard-wired operation of the human system for recognition and identification, victims of the “rush to the object” (to use Michael Baxandall’s apt term; Baxandall, 2003, p. 130). Recognition and the resulting swift displacement, moreover, need not just relate to the visible content of the image or object, but, depending on the level of expertise and the viewer’s ability to categorize, might rather involve the recognition of a style or an author ^3^.

Different works of art naturally present different types of constraints. On the one hand, some of them are perceptually incomplete or unstable depictions – such as many examples of modern art, whose very identity depends on the active inferential involvement of the viewer (Gamboni, 2002). The challenge of resolving visual ambiguity and understanding what the painting or sculpture represents, makes the viewer aware of the interpretive process. While some aspects of the labor of this filling-in might be unconscious, it often also requires a conscious effort from the viewer, in which the process of making sense of an image is brought fully into awareness. In many artistic styles, on the other hand, the representational content is recognized and identified swiftly (in the viewer’s phenomenology instantaneously), without him being aware of any effort ^4^. In such a case the beholder’s share consists of the unconscious deployment of perceptual-cognitive routines that render the recognition of visual object. Perceptually unstable or otherwise challenging images generate prediction errors related to the recognition and identification that the viewer (as argued by Van de Cruys and Wagemans, 2011) may find challenging and rewarding to resolve, much like ambiguous perceptual occurrences in natural vision. The problem, as just described, is that once the viewer arrives at a certain “solution” (with or without some external source of information, such as a label), there is often no need to go any further beyond the recognition of content and subject and to engage in a prolonged experience. This has been repeatedly observed in empirical studies of museum visitors. For instance, psychologists who were commissioned to undertake one of the most comprehensive empirical studies of aesthetic experience wrote in their book: “*Most people, when confronted with a work of art, simply do not know what to do. Without a goal, a problem to solve, they remain on the outside, unable to interact with the work*” (Csikszentmihalyi and Robinson, 1990, p. 83).

In many (if not most) encounters with works of art, the beholder’s share thus consists of sensory and conceptual predictions which are generated and resolved at the level of scene/subject recognition and identification, without proceeding to higher levels of cognitive and emotional predictions. Predictions, that is, *expectations* about the immediate sensory environment are transferred to the perception of works of art, without any adjustment being made to expectations about the *special kind of sensory environment* that works of art (and museum or gallery setting) demand and present for the benefit of the viewer. This leads to two alternative interim conclusions. The more radical (and skeptical) one suggests that the PEM account of real-world perception, which involves “explaining away’ the driving (incoming) sensory signal by matching it with a cascade of predictions pitched at a variety of spatial and temporal scales” (Clark, 2013, p. 7), is incompatible with a deep experience of a work of art, as this kind of object resists being simply ‘explained away.’ Accordingly, the basic tenet of the Bayesian framework that the brain minimizes unpredictability is at odds with the ontological nature of works of art, which defy predictability. The less radical and more accommodating option suggests that the deeper experience of works of art requires that viewers be able to tune or adapt their prediction mechanisms to the specific visual environment of the museum and that they have the ability to create or temporally sustain a productive flow of predictions across hierarchical levels, from low-level sensory to abstract semantic and conceptual levels. It demands that viewers consciously or implicitly come to realize that the objects in artistic representations are not like natural objects, that they are depictions, which means that they are mediated by the representational structure of a given artistic medium ^5^.

## CASE STUDY

**FIGURE 1:**
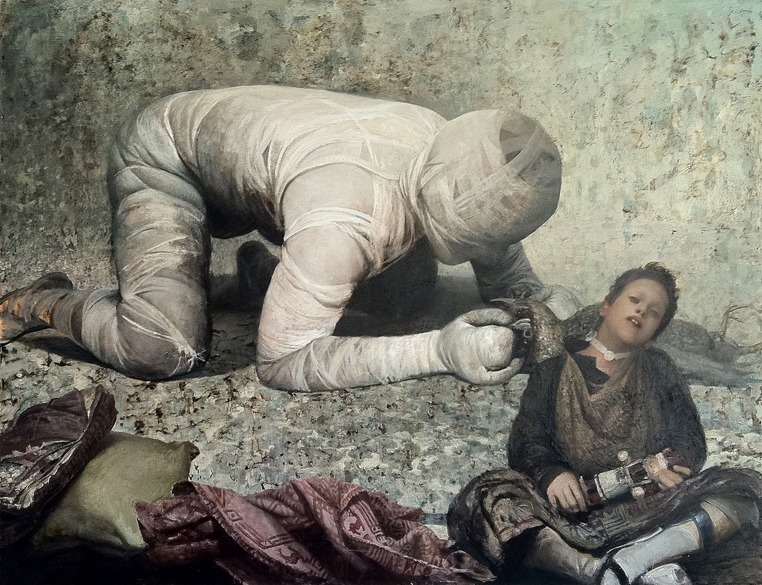
**Vincent Desiderio, Spiegel im Spiegel (2010), oil on canvas.** © Vincent Desiderio, courtesy of Marlborough Gallery, New York.

To obtain a better idea of how predictions operates in art perception, it is necessary to support a theoretical model with at least a minimal phenomenological account of actual viewing experience of specific work of art. I shall do this with a remarkable painting by contemporary American painter Vincent Desiderio, which is an analytically rewarding case of an image that is not perceptually unstable, so the viewer is able to swiftly recognize the depicted objects; however, identification does not yield to understanding in the sense of a well-understood subject or an established symbolic/iconographic theme. The viewer’s (I assume a motivated viewer, willing and able to endure more than a fleeting encounter with the painting) initial response to the picture within PEM is plausibly explained by the mechanisms by which sensory predictions subserving recognition are exercised. In addition to contextual modulation, the low spatial frequency information in the image that encodes its gross properties triggers object and category information, which in turn serves as a prediction template to guide further sensory processing, i.e., the high spatial frequency perception that conveys details (Bar, 2003, 2009; Rauss et al., 2011; Panichello et al., 2013) ^6^. But despite the relative ease with which most individual objects in a pictorial space can be identified, the beholder is left puzzled as to what is transpiring in the depicted scene, what the meaning of the painting is. There is no help to be obtained from applying the usual strategy of seeking external guidance, for looking at the caption and learning that the title of the work is *Spiegel im Spiegel* (**Figure 1**) offers no explanation or definite clue. The opacity of the painting in terms of its recognizable meaning will lead the motivated viewer to further attempt to minimize prediction error by engaging in an active search, and doing so by using both general strategies of PEM at the same time: by changing sensory input through action (that is, performing an active visual search in front of the painting) and by alternating the predictions through perception, that is, making the model fit the sensory input (Hohwy, 2013). Without a high-order generative model or predictive “template” (Summerfield et al., 2006; Summerfield and Egner, 2009) against which to match the observed sensory data, the viewer will try both to adjust his expectations and simultaneously to explain away those visible aspects that resist imminent understanding. While most objects in the pictorial space can indeed be easily identified as such, there are mismatches waiting to be resolved – most notably understanding the expressions and gazes of the faces of both figures, or precisely defining the area in which they come closest to each other.

Prolonged engagement with the image unleashes a cycle of PEM, which enters conscious awareness and may be verbalized (and even socialized if the viewer interacts with a companion) as, for example: what exactly is the bandaged man clutching in his hand? What is the function of the white band around the boy’s neck? In particular, what do the figures’ expressions convey? What state is the boy in – is he sleeping, comatose, dying? The content of the pictorial scene at the level of individual objects will be almost completely resolved as observed information is “iteratively reconciled across multipe levels of visual processing hierarchy, resulting in a progressive reduction in prediction error as the visual system settles on a single perceptual interpretation of the sensory input” (Summerfield and Egner, 2009, p. 406). However, the identification of individual objects does not directly enable inferences about their relationships and hence about the meaning of the whole scene. It thus triggers a succession of higher-level, semantic predictions, which unfold through an ongoing exploration of the painting and concern above all the nature of the interaction between the depicted figures (is their enigmatic implied relationship indeed what the painting is “about”?). Similarly, the representational status of the scene remains uncertain: is it to be perceived as a real scene, or as a fantasy image (dream, vision) of the artist, or is to be seen as the state and content of the momentary state of consciousness of one of the depicted protagonists? Moreover, for an attentive viewer, the process of prediction error minimization does not transpire just at the level of (virtual) depicted objects and their relationships. Rather, there is a concomitant awareness of how objects arise from the painterly medium – in this case from Desiderio’s rich and deeply textured brushwork, with individual marks oscillating between representational, mimetic and non-mimetic function. On that level, one of the mismatches relates to the perception of the spatial setting and its uncertain representational status. The ambiguous spatial construction thwarts and frustrates attempts to recognize it in terms of some kind of empirical environment; the viewer’s perception of the virtual pictorial space as some sort of non-descript enclosed space, delineated by the ground and the wall, easily shifts to an awareness of the picture plane and markings, which seem to deny any claim to mimetic optical veracity.

## AFFECTIVE PREDICTIONS

The entire process of individual meaning-making out of this harrowing (others used even more expressive terms such as “strikingly frightening and nightmarish”) painting, within the predictive coding framework, is steeped in affective significance. While individual feelings will naturally differ, a central part of a viewer’s encounter with the image is his or her emotional reaction to it. Recent models in affective neuroscience insist that affective meaning is not something superadded on the perceptual act pure and simple; rather, while the brain is engaged in object recognition, it concurrently extracts affective value from the observed scene, particularly its valence (Russell, 2003; Barrett and Bar, 2012; see also Jaspers, 1913/1963, p. 255 for an early formulation). Affective (and interoceptive) predictions occur in rapid timescales and concurrently, not as a separate step, consequently conscious percepts are intrinsically infused with affective value ^7^. According to Barrett and Bar’s (2012) model, the brain’s prediction about the meaning of a visual sensation includes some representation of affective impact (or similar sensations) from the past. Moreover, the affective state of the perceiver at the moment of the initial encounter exerts top–down effects on visual processing (Anderson et al., 2011), thereby constraining the formation of predictions from the sensory level upward.

Some authors have further argued that viewers recognize the “emotional gist” of the scene, referred to as the global emotionality, whereby the scene can be rapidly identified as positive, negative, or neutral without having to explore the individual (local) features of the scene. In the given case, the phenomenology of the viewing experience does not support the notion of instantaneous emotional gist, but rather that of an affective reaction that unfolds throughout the entire duration of seeing. Its initial stages appear to be related to the perception of affective affordances within the pictorial space, especially bodily postures and facial expressions of both depicted figures ^8^. As recent eye-tracking studies have demonstrated, the eye initially tends to fixate on emotional objects rather than more salient, neutral ones, and emotional saliency can override visual saliency defined by features such as intensity, color and orientation (Humphrey et al., 2012; Niu et al., 2012). At the same time, people process the emotional implications of biologically emotional stimuli related to survival or reproduction automatically, but engage in more elaborative processing when confronted with socially emotional stimuli (Sakaki et al., 2012).

The generally weak correlation between emotions and their predicted expressions (Fernández-Dols and Crivelli, 2013) is in this case further augmented by the fact that neither figure’s expression can be quickly interpreted, as they are both partly obscured and partly indeterminate. While neither figure constitutes emotional affordance of the kind usually used in neuroimaging experiments (that is, a stimulus with imminent threat or fear value), both are imbued with a saliency that involves the basic repertoire of emotional reactions. The boy’s indeterminate expression signals a loss of consciousness, illness, or perhaps even death – all of them possibilities suffused with a strong affective valence. Similarly, the man’s body, completely wrapped in bandages, constitutes a powerful affordance whose negative valence is linked to instinctive fears of illness, accident, or disfiguration, that is, basic emotions related to bodily harm and/or survival. However, it is not just such biologically determined associations that determine how the affective response unfolds, as the process will likely also involve memories of culturally transmitted contents. Thus the eerie feeling the bandaged human figure elicits may not derive from individual experiences of bodily harm or medical treatment (or fears thereof), but also from affectively inscribed memories of experiencing and reacting to similar representations of uncanny objects – e.g., in horror movies. One should likewise note that while low-level visual properties generally contribute to an object’s perceived valence (Lebrecht et al., 2012), this factor is even more important in the case of an art work. The affective salience of the figures is thus inherently constituted by the painterly medium, that is, by the way Desiderio’s brushwork and handling of color depict them as objects in the pictorial space. Furthemore, affective affordances are contextually pre-tuned (Todd et al., 2012); in art perception such pre-tuning is significantly influenced by the general experiential context (e.g., one’s feeling about being in a museum or gallery, the presence/absence of “museum fatigue”), as well as by the affective state engendered by cumulative past experience of viewing art works.

To sum up so far: in the motivated viewer’s encounter with *Spiegel im Spiegel* there occurs a complex interplay of predictions that span every level within the hierarchical structure of the mind/brain, from sensory/recognitional to high-level semantic predictions. But whereas in a real-life situation, perception, cognition, and action are associated with the successful suppression (“explaining away”) of prediction error or the reduction of surprise (Clark, 2013), the viewer’s encounter with Desiderio’s painting will almost inevitably result in a subjective awareness of not being able to arrive at an acceptable solution as to what the painting represents, what it “is about.” At the end, the viewer has to settle with the best solution for the moment; in other words, the process of optimizing relative precision of empirical (top–down) priors and (bottom–up) sensory evidence (Friston, 2009) temporarily subsides, only for the viewer to be aware that the solution is precarious and provisional. Uncertainty about the meaning, as a few available records suggest, is accompanied by an unspecified, but powerful feeling, which can be summed up in such words as uncanny, troubling, depressing, or nightmarish ^9^. This overall affective response is related both to the perception of pictorial content and the medium (as discussed above) and to the affective component of prediction error minimization itself, as we shall presently discuss.

## THE LIMITATIONS OF THE DOPAMINERGIC REWARD ACCOUNT

According to Van de Cruys and Wagemans (2011) tentative prediction error hypothesis, it is the incompatibility (prediction error) that causes a part of the emotionality that viewers encounter in works of art. Their main thesis is that the reduction of unpredictability is experienced as positive and pleasurable. Thus: “The effort of mental work one has to do to cope with the prediction error is a condition sine qua non for receiving perceptual pleasure of a Gestalt formation (prediction error reduction)” (TPE, 1046). The authors suggest that the degree of mental effort viewers make to compensate for unpredictability is related to reward. This is then further linked to dopaminergic reward modulation, whereby unexpected reward are associated with increased dopamine peaks. Artists are thus in the business of postponing the final gratification: by using minimal prediction errors painters can ensure that viewers will obtain their reward and not give up prematurely. Similar claims have recently been made by Kandel (2012), who argues “that the response of dopaminergic neurons to anticipated pleasure may be the physiological basis of the pleasure we experience when looking at art. Art may give rise to feelings of well-being because it predicts biological reward, even though further reward beyond the pleasure of viewing and vicariously experiencing may never materialize” (Kandel, 2012, pp. 428–429), and other recent opinions concur that positive emotional valence, or pleasure, is elicited in the transition from a state of high to low surprise (Joffily and Coricelli, 2013).

These views align with recent research on the neurobiology of reward. It is well-established that reward has a direct, non-volitional impact on perception, changing the salience of objects for attention (Hickey et al., 2010). Dopamine receptors were found to mediate prefrontal control of signals in the visual cortex (Noudoost and Moore, 2011). Studies based on the monetary reward prospect paradigm reveal that reward leads to the tuning of sensory neurons and modulates the neural dynamics of early visual category processing (Apitz and Bunzeck, 2012; for an overview of reward-related modification of sensory processing in the cortex, see FitzGerald et al., 2013; Overton et al., 2014). More specifically, Biederman and Vessel (2006) proposed that the interpretation of a novel and richly stimulating visual pattern leads to feelings of pleasure, because such patterns initially activate an abundant set of associations in the ventral visual pathway that manifest dense mu-opioid receptors. Such neurobiological accounts are supported by psychological research that suggests that ambiguity in works of art may be pleasurable (Jakesch et al., 2013) and collectively these findings lend scientific support to old insights that perceptual interpretation in itself is rewarding, something that is summed up in Gombrich’s (1982) observation on “pleasure of recognition.” However, as I shall argue, there are grounds for a much more cautionary approach in postulating links between reward and pleasure in visual art perception.

First, the pleasure of recognition (or perceptual Gestalt formation) should be considered a kind of cognitive reward, which differs from more basic reward (Schultz, 2000); while acknowledging the biological basis of desire for or liking of art, it should probably be distinguished from incentive salience (“wanting”) as a specific form of Pavlovian-related motivation for reward (for such distinctions, see Berridge, 2012). It is as yet unclear to what extent cognitive reward share neurobiological mechanisms with basic forms of reward, those which for instance activate a craving for food or a drug. Second, conceptualizing reward in relation to art experience in the terms of perceptual pleasure is too narrow, as it accounts for a distinct minority of encounters with visual art. If phenomenology is to be taken as a reliable guide, pleasure may indeed characterize the nature of the viewing process in the case of works in which perceptual recognition/identification is more complicated – as in the case of many modern art styles. Upon encountering Pablo Picasso’s *Guitar and Violin*, or Lyonel Feininger’s *Sailboats,* recognizing the objects in the depicted scenes – that is, perceptual prediction error minimization *tout court* – clearly is related to pleasure for many viewers, as Van de Cruys and Wagemans (2011) propose. In most instances, however, pleasure may have a rather limited – if any – role in the reward that is associated with viewing art works. A more inclusive account thus needs to reflect the recent conceptualization of reward, which sees it as separated into several components at the psychological and the neurosystemic level – pleasure, incentive motivation and learning (Dickinson and Balleine, 2010; Smith et al., 2012; Miller et al., 2014) – or alternatively insists on dissociating motivation from reward.

Finally, it should be briefly noted that the role of dopamine in mediating perceptual (or broadly aesthetic) pleasure is not very clear. The authors of one recent study confirmed the presence of dopaminergic activity during the anticipation and actual experience of peak emotion in music (Salimpoor et al., 2011), but there is little to suggest the same mechanism operates in the perception of a visual medium as well. The authors of this and other studies (Vuust and Frith, 2008; Schaefer et al., 2013) point to the significant role of temporal phenomena such as expectation, tension, surprise prediction, and anticipation in evoking emotion from music. In contrast, although perceiving painting or sculpture is likewise a temporal event, the role of expectation and anticipation is of a different order. On the other hand, cognitive wanting was found not to be directly affected by mesolimbic dopamine fluctuations (Wassum et al., 2011) and much evidence was accumulated against the long-standing view that dopamine mediates pleasure (Berridge, 2012, p. 1131).

## GRASPING MEANING – THE OUTCOME OF PREDICTIVE ERROR OPTIMIZATION

As discussed above, in many (if not most) encounters with works of art, the recognition of the subject is accomplished instantaneously and does not lead to extensive engagement with the work, that is, once the minimization of prediction error at the level of object recognition concludes the viewing, there is no expectation of further reward and hence no motivation for a prolonged viewing and thinking about the work. Returning to the case study of Vincent Desiderio’s painting, two distinct patterns of response can be postulated. In each case, the viewer, having more or less effortlessly accomplished the recognition of objects in the scene, is left puzzling over the meaning of the painting – the image itself does not provide sufficient clues with which to optimize prediction error. For viewer A, the semantic opacity of the painting does not constitute a challenge to be engaged with, resulting in a negatively valenced experience, which provides no incentive for further viewing (or for repeating such an experience). The well-documented aversion to modern art can be partly explained within this framework. On the other hand, viewer B, in the course of a much more extended, consciously reflected, viewing, experiences a cascade of prediction errors minimizations, which entails the simultaneous formation of new predictions, and thus again arrives at no final “solution” as to the meaning of the depicted scene. At a certain point she leaves the painting with the best interpretation available at the moment; in the terms of PEM, she explains away the image (and her own reaction to it) given her continuously updated generative model. The optimization of prediction error concludes with the best possible outcome for the moment, but that outcome remains tentative, as subjectively the painting retains its enigma, lingering in memory and even generating new associations. The experience itself, although subjectively felt as something disquieting, troubling and certainly not inherently pleasurable, may ultimately be perceived as rewarding, and as providing motivation for another encounter of this kind. This fully accords with some recent accounts of aesthetic experience as being disruptive and transformative at its core (Pelowski and Akiba, 2011) ^10^.

This is not the end of story, however, as our case study provides an apt opportunity to observe how the response to art work within the PEM framework is further, and perhaps decisively, affected by the viewer’s access to external facts, some kind of extra-pictorial information that cannot be gathered from the visible configuration of the image itself. In the given case, the key information is the knowledge that in this (and several other paintings) Desiderio depicted his severely physically and mentally handicapped son Sam, who needs a tube to breathe and whom he has been constantly caring for. The viewer realizes that the painting is not to be understood entirely as a fantasy image or a dream, and that the visible content of the scene refers to an existing aspect of reality. Some remaining sensory mismatches are consequently minimized (“the white tube around the boy’s neck is the breathing tube”), while simultaneously new (semantic) ones are generated (“If the boy is the artist’s son, is it likely that the bandaged figure is the painter himself? Is the whole painting, then about the artist’s (a father’s) relationship/communication, or rather about the inability to communicate with his gravely disabled child?” A brief quote from comments made by an exceptionally perceptive art critic captures eloquently the range of associations and feelings that may unfold for a perceptive viewer, and the following, in fact, is a verbal transcription of the process of PEM unfolding in the critic’s mind: “*Empathically identified with him, and bent over him in imploring care, Desiderio is unable to establish intimacy with his son, all the more so because he seems lost in an dream world of his own. His son will remain a child in spirit and body all his life. Damaged beyond repair, the boy will never become a man. I know no greater image of human suffering in contemporary art, no subtler image of modern alienation – the absurd nightmare that is modern life*” (Kuspit, 2011). Most viewers may not be able to form as elaborated and sophisticated an association as an experienced art critic, but their understanding of the representational content of the image will nevertheless alter the profile of their affective response, in which the unspecified negative feeling, as described above, can be replaced by empathic engagement with the figures depicted. This empathic reaction will in turn entail both affective resonance and cognitive perspective-taking based on generating imaginary scenarios, that is, it will involve both cognitive and interoceptive (Seth et al., 2012; Seth, 2013) predictions about what it would be like and how it would feel to be in the same situation. Therefore, reducing the uncertainty related to the representational content of the painting by minimizing prediction error, far from “explaining the painting away,” decisively changes the generative model of the motivated viewer, thus triggering a new productive cycle of perceptual, cognitive, and affective inferences.

## PREDICTIVE ERROR MINIMIZATION AND THE CULTURALITY OF VISION

In this section I shall point to some strategic implications of the predictive coding framework for art history and visual studies. The observation that predictions in visual art are dependent on the specific history of stimulation (Van de Cruys and Wagemans, 2011, pp. 1044–1045, see also Clark, 2013) corresponds to the long-standing and widely shared understanding that perception depends on one’s personal and cultural background (Arnheim, 1954; Segall et al., 1966). Recent research has extended these views by examining the qualitative difference in the ability to predict between experts and novices, emphasizing that experts have more resources for generating predictions, but also that they make more elaborate and accurate predictions in a given context (Cheung and Bar, 2012; Panichello et al., 2013). But importantly, the inter-individual differences in art perception do not stem from expertise alone, but need to be conceived more broadly. Summarizing earlier insights, Gombrich (1960) argued that perceptual experience depends on “mental set,” without much elaborating of this notion. More precisely, as I shall argue, the inter-individual differences in art perception depend on three variables: (i) personality traits/affective style – that is, how and why individuals differ in how they respond to emotional incentives (Davidson, 2004). These have a strong modulatory effect, especially with respect to the affective aspects of art perception, so, for example, individuals with neurotic and anxious personality traits are more sensitive to processing facial or bodily expressions in particular (Bishop, 2007; Cunningham et al., 2010; Cunningham and Brosch, 2012); (ii) culture-cognitive capital related to the experiential situation, that is, the skills and knowledge related to visual perception and viewing art works; and (iii) the momentary psychosomatic state of the observer.

Jointly, these three aspects form a mindset, which determines the generation of top–down expectations and predictions. Alternatively, mindset itself can be conceived of as the sum total – or repertoire – of predictions that pertain to the given task (Bar, 2009) and as such it is further primed by the given experiential situation. In the case of a typical visual art experience, entering the museum or art gallery (or mere prospect thereof) serves to prime the mindset, forming a global expectation about the experience – a potentially fascinating and enjoyable event for person A, or the prospect of something boring and tedious that has to be endured for person B, with many variations in between. This general expectation conditioned by all three variables thus sets the stage for specific predictions to be generated vis-a-vis the individual works of art encountered during the visit.

Importantly, experience-based individual differences in viewing art works, which are partly dependent on culture-cognitive capital, link the predictive coding account of art perception to a major issue in art history and visual studies – the problem of the culturality and sociality of vision. In these and related disciplines, the biological-social continuum of seeing is routinely conceptualized as a distinction between vision (as a biological act) and visuality (as culturally and socially determined; e.g., Nelson, 1996; Davis, 2011). As recently articulated by Davis (2011, p. 230): “When we speak of visuality, rather than simply vision or visual perception, we address the difference introduced into human seeing by traditional cultural meaning consolidated and reconfigured in images.” This conceptual distinction, however, is problematic and calls for alternative framework, which would have to consist of four levels in order to capture the process of vision in its biological and social complexity with more precision (Kesner, 2009, 2014). Moving in a top–down fashion, these are the levels of:

(1) Concepts, attitudes, values, and motives (and their discursive articulation) about images, vision and representation— that is, visuality in the strict sense of the term*;* these develop and persist on a time-scale of years to centuries. (2) The level of cognitive factors, which is strongly shaped by the environment and culture and roughly corresponds to Baxandall’s (1972) notion of the “period eye”: semantic categories, patterns of inference experience and training in the range of representational conventions etc., that is, factors that operate in stretches ranging from the minutes of psychological time in individual perception to the historical time of years. (3) Perceptual strategies and processes – such as mechanisms of recognition, object identification and classification, patterns of saccadic eye movement, of selective visual attention, processes of unconscious embodied emotional and emphatic response, motor reaction activated by perception etc. These are processes that operate on both the conscious and the unconscious level and span a time frame of 100s of milliseconds to minutes. (4) Mechanisms of detection of essential aspects of the scene, such as lines and edges, movement, color, binocular disparity, and related aspects of low-level vision – that is, biologically hard-wired unconscious events occurring on a time scale of up to ∼250 ms. Naturally, such a scheme implies neither a strict hierarchy nor hard boundaries between these stages, nor their mutual encapsulation. To the contrary, there is an ongoing, reciprocal relationship and feedback, whereby biologically embedded mechanisms interact with the higher levels of vision that can be modified by culture. Prediction errors at level 4, i.e., low-level vision, concerning mainly contrast and orientation, are sent further on in the processing stream and integrated into more complex messages concerning object identities, and then further on into semantic categories. The two topmost levels – of visuality and cognitive categories – are in a strong sense culturally relative. But importantly, there is an increasing body of evidence suggesting that also at level 3, concerning perceptual strategies and processes, both culture and individual perceptual history penetrate perception (for an overview of this evidence, see Kitayama and Uskul, 2011; Rule et al., 2013). These stages operate on different time-scales and to an extent can be mapped as occurring in different areas of the brain, where higher levels represent the context in which the lower levels unfold (see also Kiebel et al., 2008; Hohwy, 2013, p. 61). Representations thus depend on and interact with representations at other levels both within the topography of the brain and within the hierarchical conceptual scheme of vision outlined above.

The experience-mediated perceptual-cognitive routines and skills that ensue from a viewer’s participation in a shared sphere of cultural habits and protocols of seeing and a sensitivity that is attuned to styles of representation ingrained in the viewer’s culture are all the produce of perceptual learning and – at the neuronal level – the mechanism of synaptic plasticity. There is an extensive body of recent research providing evidence on experience-dependent plasticity in adult brains and specifically on how perceptual expertise alters visual processing, e.g., by determining nature of object representation in the visual system (Curby and Gauthier, 2009; Spolidoro et al., 2009; May, 2011; Wong et al., 2012; Folstein et al., 2013; Lövdén et al., 2013; Kok and de Lange, 2014; Vetter and Newen, 2014). It has been shown that the integration of top–down expectations and bottom–up sensory input can already be observed in the early visual cortex (Kok et al., 2013) and that past experience modulates shape assignment and perceptual grouping (Kimchi and Hadad, 2002; Trujillo et al., 2010). Cast in the terms of the PEM framework, previous experience and perceptual expertise generate distinct set of expectations (or priors) which determine interpretation of the image. Formation of a prediction error is achieved by adjusting synaptic efficacies both between and within levels of the processing hierarchy (Schultz, 2000; Friston, 2008). Post-synaptic effects either may be short-lived, directly impacting perception, or may control the updating and storage of predictions by inducing changes in synaptic growth (den Ouden et al., 2012). It is likely that it is by this mechanism that perceptual expertise becomes stabilized in the individual mind and by which it can even become collective in the sense of characterizing the perceptual habits of a certain group of people, so that commonalities can be observed in the social world of a particular group of viewers that govern how they form predictions in the perception of works of art (“period eye” according to one influential art-historical paradigm – cf. Baxandall, 1972).

This kind of expertise is both enabling and constraining, as the following example will show. Chinese literati and literati painting form a well-defined group of expert viewers of a well-defined body of art that to be understood requires a strong and specific form of visual-cognitive skills, some aspects of which can be indirectly inferred from their extensive writings reflecting on the subject. The predictions involved in their experience of painting operated at all four levels of the hierarchy: at the level of visuality, value-based judgments and preferences suggested what was worth looking at and was deemed to be of aesthetic value for the given social group and this determined the most general parameters of the perceptual encounter (e.g., by constraining attentional allocation according to the perceived value of the painting). At the level of cognitive factors, literati culture shaped predictions related to the semantic categorization of paintings, thus enabling the viewer-expert to differentiate and conceptualize not only specific topics and subjects or various styles, but also the variety of brushwork seen in the painting (so-called *cunfa* brushstrokes). Finally, on the level of perceptual routines, distinct patterns of visual attention shaped by experience, along with saccadic eye movements or an embodied kinesthetic proprioceptive feeling of brush wielding determined the formation of predictions related to the imminent perceptual processing of these paintings.

This expertise, comprising a vast store of memory representations and semantic associations, but also internalized motor representations and body schemas, provided the expert viewer with the ability to unfold successive strings of prediction errors, from which a rich set of expectations could be formed, entertained and finally minimized. In contrast, the naive viewer, lacking such cultural equipment with which to approach this particular kind of art work has at his disposal a far more limited repertoire of resources to respond and create meaning. Note, however, that this type of culture-specific expertise attuned to a specific form of representation was also constraining, as the insider-viewer was equipped with a fixed set of routine and rigid associations, generating expectations and predictions that shut out some possible other ways in which these works could be perceived- hence the well-documented narrow focus of literati painters on the quality of the brushwork in a painting, while ignoring the representational quality and mimetic status of the image. The above example would typically have involved a continuous bidirectional flow of vision across the hierarchy of levels, from high-level predictions (visuality) down to the level of basic perceptual mechanisms and routines, and vice versa. If culture can be seen as generator of hyperpriors, essential to the hierarchical network of the Bayesian brain, which is the source of a person’s perceptual fantasy of the world (Patton et al., 2013), the crucial task for understanding this is ultimately to provide a naturalistic account of how both innate and learned priors, or contextual and structural expectations (Seriès and Seitz, 2013), or informed vs. fixed priors (van Wassenhove, 2013) arise and are mutually co-determined and modulated.

A likely candidate for the central, enabling hyperprior ^11^ operating in art perception is the general pictorial competence and the capacity of seeing in/as, that is, the ability of the human observer to see a certain object in a depictive configuration on a pictorial surface, to see “through” the depiction to its referent and at the same time to the nature of the relation between representation and referent. This capacity, as psychological research demonstrates, is acquire *de novo* in infants and develops ontogenetically in humans, and it can thus be considered universal (DeLoache et al., 2003; DeLoache, 2004; for a historical perspective on the role of seeing in/as in the origins of picture-making see Davis, 1986, 2011). But as such, it is decisively modulated by many culture-specific effects, which include a range of representational conventions, the nature of a particular visual medium and many other factors spanning the hierarchy of vision from visuality to perceptual routines. It would be enormously complex to formulate a fully naturalistic account of how hyperpriors, co-constituted at the intersection of biology and culture, enable and constrain the formation of predictions when viewing particular art works. But importantly, some aspects of it are open – at least in principle – to experimental study. As a brief example, consider the case of the perception of a visual symbolic form by the excessive action computer-game players. In this well-defined group of image users, research has provided empirically measurable evidence of the modification of certain perceptual routines (e.g., Boot et al., 2008; Donohue et al., 2010; Bavelier et al., 2011) and even of players’ affective responses to imagery (Montag et al., 2012). One way of broadly summarizing these findings may be that the habit of on-line acting upon, motorically responding to a stream of constantly changing images resculpts the perceptual-cognitive architecture of these players on a synaptic level, ultimately giving rise to specific variations in their general pictorial competence – a hyperprior expectation that images are entities to be acted upon instantly, not something to be perused and contemplated in a prolonged fashion. The explanation for how such individual, experience-dependent expertise and plasticity characteristic of this kind of temporally delimited collective visual experience may become stabilized, more permanent and transmitted to successive generations within the same cultural milieu remains a major desideratum for further research, but this is a topic beyond the scope of present article.

## CONCLUDING REMARKS AND FUTURE DIRECTIONS

I shall conclude by briefly outlining several possible directions in which the present accounts of the predictive coding framework for the visual arts can be further productively elaborated. The first direction, mentioned above, concerns the possibility of making some key theoretical accounts in art history and visual studies – such as Gombrich’s (1960) classic account of the rise of naturalistic depiction, or “a general theory of visual culture” (Davis, 2011) – compatible with the prediction error minimization framework. The second direction is to elaborate the model on the basis of further case studies of specific types of visual art objects. The theory could likely be productively applied to many pre-modern works of art, which in their original context of use served as objects endowed with specific functions, and where the “viewing” in the original setting was inextricably bound up with (or accompanied by) some sort of embodied action. Such works can be said to contain their own script for action; the experience of the original audience could thus be modeled through action-oriented predictive processing (Clark, 2013), which suggests that motor intentions, as they unfold into detailed motor actions, actively elicit continuous streams of sensory results that our brains predict. Furthermore, while Van de Cruys and Wagemans (2011) note that the predictive framework does not explain the popularity of realist art, “which depicts the world as it is, thereby confirming rather than violating prediction error,” (TPEA, 1056) this genre of painting need not be discounted and the PEM framework can be elaborated for the naturalistic/realistic spectrum of artistic representations as well ^12^.

The third challenge is even more complex, but offers the potential for truly interdisciplinary dialog between theorists of art and neuroscientists. It has been argued that the actual virtue of predictive coding is the fact that it is typically implemented at a level of abstraction that is intermediate between that of low-level, biophysical, circuits and that of high-level, psychological, behaviors (Spratling, 2013). While this indeed seems to be the case, the greatest challenge (as noted in the opening section) is linking the accounts of predictions at the neuronal level with those on the cognitive-psychological and cultural levels (see also Clark, 2013). While current research is providing an increasingly detailed insight into neuronal mechanisms, including an account of the interactions between prediction and error signals (Kveraga et al., 2007; Summerfield and Egner, 2009; Rauss et al., 2011; Friston et al., 2012a; Koster-Hale and Saxe, 2013; Kok et al., 2013), the relationship between the various levels of prediction operating in visual-arts perception, between the neuronal-architecture and cognitive-psychological levels of prediction, has hitherto been at best tentatively and sketchily explained. In particular, future work needs to address the nature of representational formats of hierarchically different levels of predictions. In other words, the notions of predictions (priors) needs to be related to range of terms currently used both within and outside the predictive coding framework to characterize disparate contents of mental representations underlying the recognition and interpretation of sensory content from across the hierarchy of vision, including, for example, the “generative image model” (Yuille and Kersten, 2006), the “pictorial schema” (Gombrich, 1960), the “image schema” (Lakoff, 2006), or “subjective internal representation” (Smith et al., 2012). Much will also depend on whether (and how) some alignment can be made between the predictive coding framework and other current accounts of image perception, such as incremental grouping theory (Roelfsema and Houtkamp, 2011).

The fourth challenge relates to determining how affective and empathic inference, as conceived in the PEM framework, arises out of integrated interaction between large-scale brain networks (Bressler and Menon, 2010; Oosterwijk et al., 2012; Barrett and Satpute, 2013). As Friston (2013, p. 1331) remarks, “the most prescient challenge to formal description of the brain as inference machines is how one can accommodate emotions.” Of prime importance we need to decipher the nature of the interaction that occurs between the affective feelings elicited by prediction errors formed as the content and medium of an image are processed and the affective valence elicited by the outcome of the process of PEM (or, in conformity with a recent conceptualization of this problem, we need to decipher the relationship between unsigned perceptual and cognitive prediction errors and signed reward prediction errors (den Ouden et al., 2012). Finally, future experimental work, building on the neuroimaging and eye-tracking work done with non-art images (e.g., Itti and Baldi, 2009) and, most notably, combining subjective, behavioral and neuronal data, may untangle some aspects of prediction in art perception in different hierarchies and their interaction. Analysis of cultural hyperpriors, on the other hand, will likely have to be performed within the territory of individual disciplines, rather than predictive coding framework alone thus necessitating a multi-level, multi-disciplinary methodology (Clark, 2013; Newsome, 2013). The challenge for the Bayesian account of art perception (and equally for the neuroscience of art research generally) lies in reconciling the demands of the subjective, phenomenological description of the experience of a work of art with the rhetoric and the argumentational style of discourse used in mind and brain science and with a formalized account of prediction error theory. Recognizing the inherent difficulty involved in meaningfully bridging these two modes of argumentation (a problem that undoubtedly surfaced in present article) I still wish to argue that real progress at the intersection of art and mind/brain science can only be achieved by attempting this kind of direct interfacing.

## Conflict of Interest Statement

The author declares that the research was conducted in the absence of any commercial or financial relationships that could be construed as a potential conflict of interest.

## Footnotes

1 Support for this view can be found in some psychological investigations that found that abstract and semi-abstract paintings were rated as more meaningful when they were accompanied by their titles as opposed to without titles. This finding is consistent with the assumption that titles carry information and this information aids the meaningful interpretation of the painting (Russell and Milne, 1997).

2 As current experiments have demonstrated, it takes no longer to determine an object’s category than simply to detect its presence (see Grill-Spector and Kanwisher, 2005).

3 Identification, resulting in the substitution of the artists’ name or particular style in place of the object itself, is likely to occur in cases of non-representational art (Hasenfus et al., 1983). Summarizing earlier findings, as well as their own empirical studies, Leder et al. (2004) argue that experts interpret art work on the basis of art-specific concepts, while non-experts or naïve viewers tend to use stereotypical responses, probably drawing upon their “everyday repertoire” of concepts, such as “personal experience, feelings and a similarity of personal surroundings” (Augustin and Leder, 2006).

4 Observers can comprehend a variety of perceptual and semantic information within the first fixation, at a mere glimpse, occurring in less than 100 ms. When consolidated, within a few 100 ms, the conceptual gist of what is being viewed can be represented in the form of a verbal description of the scene’s image, a description that includes both what was perceived and what was inferred.

5 These alternatives could be also situated within the broader question (and ongoing discussion) whether and to what extend human observers are Bayes optimal. Critics point out that the basic tenet of Bayesian models that human behavior can be explained solely in terms of optimal probabilistic inference in any given situation is inadequate for explaining psychological phenomena (see e.g., Jones and Love, 2011; Bowers and Davis, 2012). Others, however, argue that humans are not “Bayes optimal” in an absolute sense (Clark, 2013, p. 9), but relative to the immediate context of the situation.

6 However, Panichello et al. (2013), in their overview, add a cautionary note: “Given that the specific neural processes that give rise to conscious perception remain unclear, it is difficult to conjecture precisely how predictive feedback influences the contents of awareness.”

7 Barrett and Bar (2012) note that the affective value of the object is sometimes represented as a property of the object, and other times it is represented as a person’s reaction to that object.

8 I use the concept of affective affordance in much the same sense as the notion of affective salience, defined as “the tendency of the item to stand out relative to its neighbors due to an association between the semantic meaning and emotional arousal” (Todd et al., 2012).

9 I derive this observation both from the few available comments on this painting and from ten interpretations of it written by participants in my graduate seminar on ekphrasis and the description of works of art.

10 Van de Cruys and Wagemans (2011) note that people may experience positive emotions when faced with an “unsolvable painting” by reappraising negative prediction error in a safe context, or, alternatively, by misattribution (TPEA, 1053).

11 Following Newsome (2013), I take hyperpriors to refer to general facts, or expectations, about “embodied interaction with the physical world that structures and constrains prior predictions and how they interact.”

12 As one of the reviewers of this paper suggested, realistic art heightens the tension between inferring the depicted object as a cause and painterly medium as a cause. Either interpretation gives a predicition error, leading to alternations in perception.
